# Open, reproducible hardware for microscopy

**DOI:** 10.1098/rsta.2023.0112

**Published:** 2024-06-03

**Authors:** Richard Bowman, Caroline Müllenbroich, Benedict Diederich, Gail McConnell, Sanli Faez, Julieta Arancio

**Affiliations:** ^1^ School of Physics & Astronomy, University of Glasgow, Glasgow G12 8QQ, UK; ^2^ Leibniz Institute of Photonic Technology, Jena, Thuringia 07745, Germany; ^3^ Strathclyde Institute of Pharmacy and Biomedical Sciences, University of Strathclyde, Glasgow G4 0RE, UK; ^4^ Nanophotonics, Debye Institute for Nanomaterials Research, Utrecht University, Utrecht, The Netherlands; ^5^ Technische Universität Berlin, Berlin 10623, Germany

**Keywords:** microscopy, reproducible science

Reproducibility and transparency have always been central tenets of good experimental science. In recent years, these have led to ‘open science’ practices, including openly archived data, openly licensed code and open access to publications describing key results [1–3]. However, instrumentation often lags behind data and results in terms of openness: there are, as yet, no widely adopted conventions requiring the designs of a novel instrument to be shared alongside manuscripts describing its use [4], though there is an increasing number of projects that do share plans for replication [5]. This special issue collects several articles that discuss examples of projects endeavouring to adopt open hardware as a means to better reproducibility, or greater accessibility, of cutting-edge microscopy. We also include some perspectives on future directions, and on how open hardware might offer an improved way to develop and commercialize novel microscopes.

This special issue is associated with a Royal Society Theo Murphy Scientific Meeting, held in Glasgow in May 2023. The meeting comprised four panel discussions with short talks from the panellists and four ‘unconference’ sessions that created space for discussion of a range of topics. These included technical topics, such as compatibility between projects, how to ensure users of technology are central to the development process and ways to ensure quality when designs are reproduced. We also considered challenges around funding and intellectual property management, and on how we can organize as a community to influence policy and achieve critical mass.

During the panel discussions, we were able to hear from researchers behind several open hardware projects: the EnderScope three-dimensional printer-based microscope [6], a low-cost optical projection tomography system [7], the M4All microscope, which included a quantitative alignment procedure [8] and the OpenFlexure Microscope [9] are all represented in this issue.

A recurring issue in discussions of open hardware is the management of intellectual property as it relates to hardware designs. Unlike data or manuscripts, hardware designs are often patentable, and universities have traditionally followed a model, whereby inventions are patented and licensed to companies. This often leads to those inventions being obfuscated or omitted when work is published, in order to avoid undermining a future patent application; this obfuscation is very much against the spirit of open science and is discussed further by Stirling in this issue [10]. Engagement with University Technology Transfer Offices (TTOs) has generally been positive, with TTOs recognizing the value of work having an impact beyond the metrics of patents and license income. However, a lack of understanding of the mechanisms and advantages of open hardware means this route is not often promoted or supported by TTOs. Since the meeting, guidance has been developed and shared, led by one of the meeting’s co-chairs [11]. The timing and scope of openness were discussed in another session, highlighting the choice between releasing work openly only when it is complete, or working in the open as a project develops [12]. The former is more usual in academia, while the latter is the norm in open software (from which there is much to learn). Clarity on the openness or otherwise of a project is important from the start, and difficulties have not infrequently arisen from differing expectations and claims of openness. This relates to the governance of open projects, which is a key aspect of ensuring long-term sustainability of particular instruments and was discussed in later sessions.

Open projects are often organized very differently from large commercial undertakings—this was most famously discussed in ‘The Cathedral & The Bazaar’ [13], which contrasted the tightly managed, top-down approach of monolithic, commercial software such as Windows with the many small projects that make up Linux. While many smaller, focused projects are often a strength, the academic system provides perverse incentives, encouraging the creation of new projects over contributing to established ones when the latter would be more efficient and valuable. There is also a need to consider how to make open projects compatible with each other, in order to allow them to be used in combination, which is how the bazaar model succeeded for Linux. The strongest conclusion reached during the meeting regarded interfaces: standards for software and hardware interfaces are useful, but very hard to agree on without a top-down hierarchy. On the other hand, even non-standardized interfaces that are well documented can enable a skilled implementer to combine different projects, and so perhaps the most important point is that we should consider how our projects might interface with other hardware and software and ensure those interfaces are well documented—with drawings, API descriptions or other specifications.

As a community, we also identified opportunities to reward good practice (such as making contributions to existing projects, or better documenting interfaces) through citations, peer review comments and other forms of community recognition. It is easy to assume that funding agencies and journals drive what is rewarded or penalized in academia, but funders and publishers at the meeting made the point that they aim to be led by the academic community. There is an opportunity for us to engage with both of these important stakeholders to spread best practice and help support people and projects that exemplify best practice—whether as peer reviewers, editors, advisers or in other roles we play within the academic system.

Another key element in making open science a standard for the future is advocacy, sharing best practice with colleagues and communicating the benefits of sharing designs openly even before a manuscript is published. Raising awareness of open-source hardware in the academic community is important if it is to become a globally accepted standard for transparency and reproducibility in instrumentation. There is still a lot to do, but the meeting showed that there is also a lot of enthusiasm outside of the community that already identifies as ‘open hardware researchers’.

As well as developing and using open hardware, there is a valuable opportunity for it to be used to make a difference. This challenges the conventional patent-and-license model used to transfer technology from universities to companies, requiring new business models and new forms of agreement. While it is not yet the default approach, scientific projects such as OpenFrame [14] and Miniscope [15], and more general projects like Arduino [16] and various three-dimensional printers [17] have demonstrated that open hardware is compatible with successful commercial production. Open hardware can lower the barrier to entry, removing patent and licensing costs and enabling small-volume production. On the other hand, the lack of exclusivity can make it difficult to justify making a potentially risky investment in launching a new product at scale. Many scientific companies are now engaging more with open hardware and software, for example, ThorLabs are making use of Arduino microcontrollers as a way to offer customizability in some products, and so the landscape is becoming less polarized between fully open and fully proprietary systems in places. Breaking into regulated markets, such as medical microscopy, is another barrier to entry, and Knapper *et al*. [9] discuss the challenges of developing open hardware to support products in a regulated market.

The in-person meeting was followed by an online event one week later, where we welcomed more participants who could not attend in Glasgow. This included sessions on software, funding and sustainability, which again highlighted the challenges of maintaining valuable open community resources when funding is generally awarded on the basis of novelty rather than ongoing value.

The meeting brought together a spectrum of participants including academic microscopists, application-focused scientists, companies, funders, publishers and technology developers. While there were many challenges identified, there was also a clear sense of community, and it is this that lends an optimistic conclusion to both the conference and this introduction. As members of the scientific community, we are called upon to prioritize funding applications, review manuscripts and nominate each other for awards. By building consensus around open science principles, we are in a position to reward good practice and to give funders and publishers the mandate they need to support open practices. We look forward to a future where science is open and reproducible by default, and invite you to read the articles in this special issue as progress towards that ideal.

## Data Availability

This article has no additional data.
